# Combined Vertebral Augmentation for Vertebral Body Fracture With Contraindications for Traditional Techniques

**DOI:** 10.7759/cureus.48517

**Published:** 2023-11-08

**Authors:** Marcos G Baabor, Bayron Valenzuela Cecchi, Gabriela Fernández, Lucas González-Johnson, Alann Peña, Hernán Delso, Pedro Vázquez

**Affiliations:** 1 Department of Neurology and Neurosurgery, Hospital Clínico de la Universidad de Chile, Santiago, CHL

**Keywords:** vesselplasty, kyphoplasty, vertebroplasty, vertebral compression fracture, spine fractures

## Abstract

Introduction and objective: A vertebral compression fracture (VCF) can be found in trauma, osteoporosis, and tumor pathology. The most frequent is the pathological fracture in osteoporotic vertebrae in the elderly. Percutaneous techniques of vertebral cementation allow treatment of A1-A2 AO spine fractures, improving pain control and spine stabilization and decreasing mobility and mortality. Traditionally, the selection of patients is fundamental for spine surgery success, with an absolute contraindication being posterior wall involvement (A3-A4 AO spine fractures) or VCF with a loss of height greater than 50%. In this report, we present a variant surgical technique combining percutaneous spine surgery with cementoplasty for patients with classical spine surgery contraindications.

Methods: Five patients with complex symptomatic VCF or A3-A4 AO spine fractures in pathologic bone with MRI short tau inversion recovery (STIR) sequence (+) were operated on with a combined technique (percutaneous kyphoplasty (KP) and vesselplasty). The visual analog scale* *(VAS) was used to measure postoperative pain.

Results: The procedure was performed within 60 days of the fracture in all patients. The mean hospital stay was two days. No patient developed major complications. All the patients had a satisfactory clinical (improvement in pain control) and radiological response at the perioperative period and at a 30-day follow-up.

Conclusions: The combined percutaneous technique allows surgical resolution of cases previously considered contraindicated, especially in elderly patients and those with comorbidities, without involving higher cost, complications, surgical time, and hospital stay. We suggest a novel, safe, and effective variation of the vertebral cementoplasty technique.

## Introduction

In elderly patients, vertebral fracture is a major cause of axial pain, dysfunction, and morbidity. Osteoporosis is the main cause of vertebral compression fracture (VCF) [1], followed by neoplastic lesions (mainly metastatic cancer and myeloma) and others such as trauma and infections [2,3]. VCF is present in approximately 4% of patients with lower back pain who consult the primary healthcare system [4]. While some are asymptomatic, other patients present with acute, localized axial pain that can be disabling. The spine is the most common site of osteoporotic fractures. Risk factors for these fractures include chronic glucocorticoid use, gender, and advanced age [3].

Among patients with axial pain in primary care, less than 1% will have a serious etiology (cauda equina syndrome, metastatic cancer, and spinal infection). The bone is one of the most common sites of metastasis [5]. A history of cancer is the most important antecedent to axial pain due to bone metastasis [6]. Among solid cancers, metastatic disease of the breast, prostate, lung, thyroid, and kidney accounts for 80% of bone metastases. Approximately 60% of patients with myeloma have skeletal lytic lesions present at diagnosis. Pain is the most common symptom, but they may also have neurological symptoms due to spinal canal compression or instability.

The importance of the problem lies in the fact that 25% of postmenopausal women over 50 years of age and at least 40% of those over 80 years of age will have a VCF [7,8]. These fractures result in 150,000 hospital admissions and more than 160,000 outpatient visits per year in the United States [7]. Although many patients will respond favorably to medical treatment (rest, analgesia, and bracing), more than 40% of patients may not achieve significant pain relief within 12 months of symptom onset [9]. It should be noted that prolonged rest for elderly patients is detrimental to their overall health. Because of this, percutaneous spinal surgical techniques of spinal cementation are often used to accelerate the resolution of symptoms and the return of early function for overall patient improvement, especially in elderly patients [7,10]. The most frequent cementation techniques are vertebroplasty (VP) and kyphoplasty (KP), recommended options to treat VCF [11,12]. The VP is a cement injection in the body of VCF, and the KP is a balloon insufflation or lifting followed by cement injection.

In this study, the authors propose a variation of the described techniques of vertebral cementation to extend its indications in a safe and effective way in patients who are not candidates because the traditional techniques of VP or KP are contraindicated, especially in elderly patients with comorbidities.

## Case presentation

This study was approved by the Institutional Ethics Committee of Hospital Clínico de la Universidad de Chile (approval no. 78). We reviewed all patients at Hospital Clínico de la Universidad de Chile, Chile, from January 2020 to January 2023 who underwent percutaneous spine surgery with the combined cementoplasty technique. All were adults with vertebral fractures in pathologic bone. In all cases, surgery was performed within 60 days of the fracture. The inclusion criteria considered the presence of pain (visual analog scale (VAS) >5), pain refractory to medical treatment, fracture (only type A3 or A4), and MRI short tau inversion recovery (STIR) sequence (+) for evaluation of traumatic changes, demonstrating recent active vertebral fracture (bone marrow edema with ongoing remodeling). The exclusion criteria were fracture A1-A2, flexion-/distraction and rotational injuries (types B and C), VAS <5, MRI STIR (-) (commonly in fractures with >60 days), medical contraindications (bleeding disorders, sepsis, etc.), and polymethylmethacrylate (PMMA) allergy.

All patients underwent surgery in the main operating room under sedation or general anesthesia according to anesthetic risk. The VAS for pain was administered before and after surgery, as well as one month postoperatively by telephone.

Representative clinical case and suggested surgical technique

**Figure 1:**
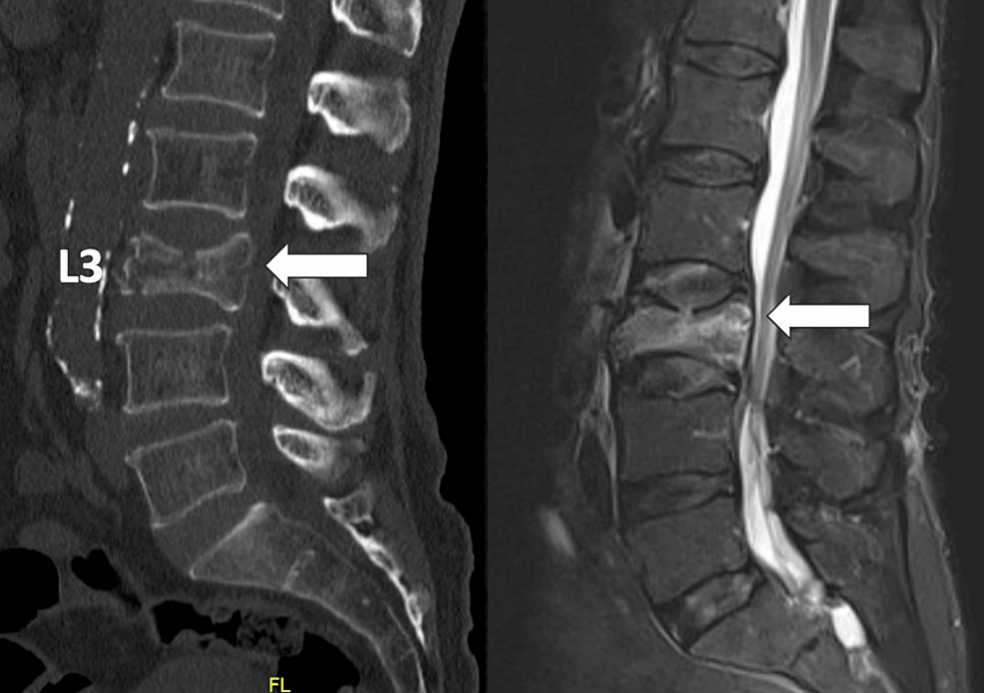
Bone window CT scan (A) and MRI STIR sequence (B) of the thoracolumbar spine. L3 with fracture of the posterior wall

A 79-year-old male, Modified Rankin Scale (mRS) 0 prior to the onset of symptoms, was admitted for severe disabling low back pain to the emergency department of Hospital Clínico de la Universidad de Chile in the context of a low-energy fall. He presented with lumbar pain after two days of evolution, disabling him with an inability to walk. The neurological examination on admission showed pain on palpation of the spinous process of L3 (VAS 10/10), without radicular signs or neurological focal signs. The Oswestry Disability Index (ODI) was calculated at 96% (maximum functional limitation). The admission images showed a fracture of the L3 vertebra with involvement of the posterior wall (A4 AO spine fracture), suggestive of trauma in pathological bone, and also showed a small fracture of the right knee and left ankle (Figure 1).

**Figure 2:**
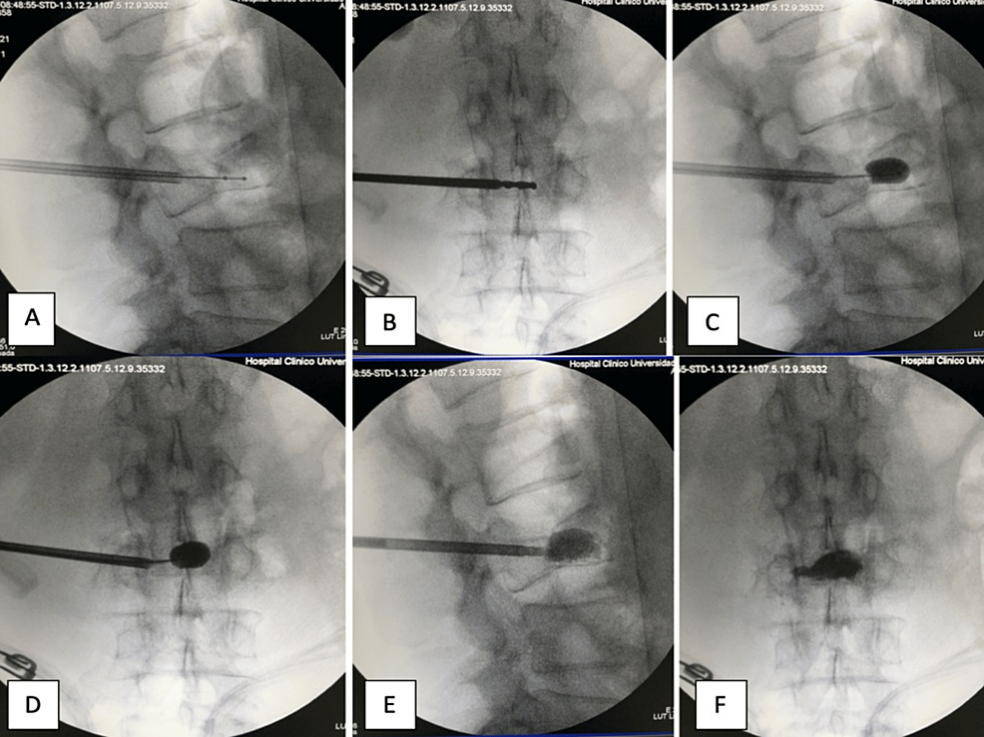
Intraoperative images. Insertion of working cannulas in sagittal and anterior to posterior view (A-B). First: KP balloon insufflation (C-D). Second: vesselplasty balloon insufflation (E-F)

**Figure 3:**
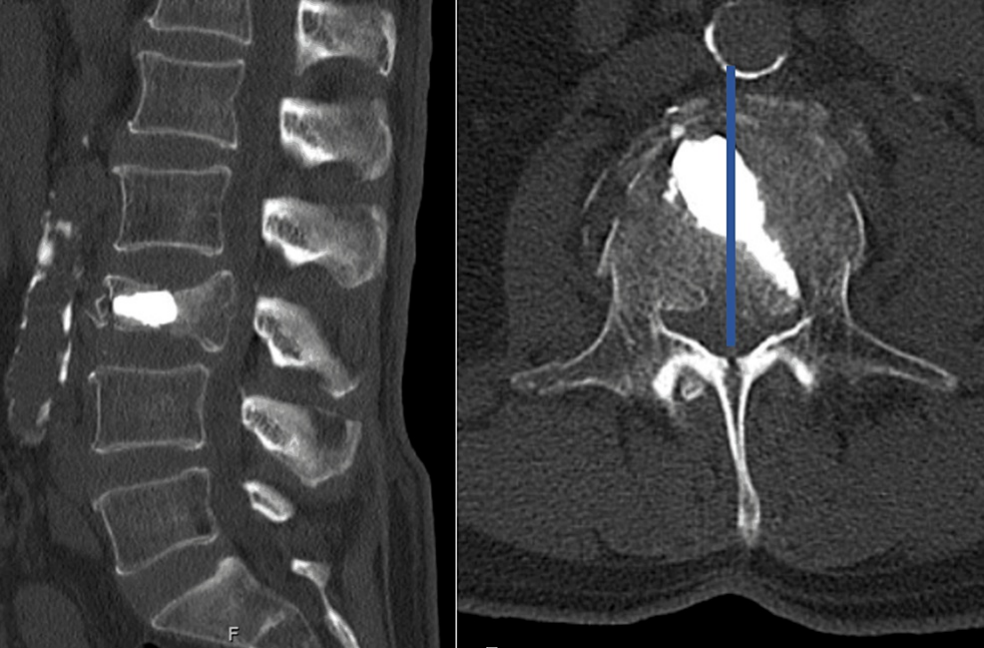
Postoperative CT in sagittal and axial view of combined technique. The line represents the midline

The STIR sequence of a complete spine MRI shows only hyperintensity in L3. He was referred to the neurosurgery service, and percutaneous cementation surgery with a combined technique for the L3 vertebra was planned (Figure 2). On the first postoperative day, the patient can sit up again and perform kinesiotherapy in bed. VAS pain was 0/10, and he was in a condition to complete trauma treatment (Figure 3). At the two-month follow-up, the patient remained with VAS 0/10, recovered previous functional status (mRS 0), and an ODI of 6% (minimal functional limitation) was calculated.

Surgical technique

Patients with painful vertebral fractures and spine MRI with T1, T2, STIR, and Fat-Sat sequences demonstrating acute and/or subacute traumatic changes and involvement of the posterior wall of the affected vertebral body (A3-A4 AO spine fracture) without spinal canal invasion were selected. Patients with type B or C fractures are excluded.

In Step 1, the procedure was performed using C-Arch Philips fluoroscopy equipment (Philips, Amsterdam, Netherlands) and sedation under local or general anesthesia. All patients received antibiotic prophylaxis in accordance with the hospital's protocol.

In Step 2, the patient was placed in a prone position. The entry space was marked at the unilateral pedicular level. Then, using a guide needle, an approach to the vertebral body was made extrapedicularly toward the midline and junction of the anterior third with the posterior two-thirds. A biopsy cannula was passed through the working channel, and a sample was taken. A precision drill was then used through the same route to create the working space in the vertebral body. Next, a KP balloon was placed to lift the vertebral body and reduce the fracture. This creates a cavity into which a prosthetic balloon will be installed. Subsequently, the Vassel-X 15 or 20 ml prosthesis (Spirit Spine, Pasadena, USA) was positioned (depending on the vertebral size), and the cement (Osteo-G, Spirit Spine, Pasadena, USA) was injected through the progressive delivery device. The injection of cement into the Vassel-X was performed in a sequential and controlled manner, allowing the complete expansion of the prosthesis (balloon or grid) inside the cavity previously created by the KP balloon, achieving the maximum expansion of the prosthesis to the limits of the cavity; periodic fluoroscopic control in anteroposterior and lateral projection was used during the whole process. This step ended when the cement came out through the Vassel-X prosthesis, which acquired a rounded morphology (Figure 2).

Finally, it is possible to visualize speculations at the limit of the prosthesis that allows for the formation of an anchorage to the rest of the vertebral body. This allows a safe and effective cementation.

Results

This case series included five patients, as shown in Table 1, which presents the demographic characteristics. The patients consisted of four females and one male, with ages ranging from 48 to 91 years and a mean age of 75 years. In this group, 80% were elderly patients. Four patients had only one fracture, and the fourth had multiple fractures, of which two were operated on, one with the classic technique and one with the combined technique. The etiology of the VCF was two cases due to osteoporosis, two cases due to low energy trauma, and one case due to multiple myeloma. The mean hospital stay was two days. The result of the surgical procedure is a significant decrease in pain in the immediate postoperative period and one month after surgery. The VAS scores in the combined technique (percutaneous KP + vesselplasty) group decreased from 9.4 ± 0.89 to 2.4 ± 0.55 one month after the procedure, respectively. Postoperative pain was significantly relieved (p<0.05) (Table 2).

**Table 1:** Clinical characteristics of the patients

	Patient 1	Patient 2	Patient 3	Patient 4	Patient 5
Age	67	91	91	48	79
Sex	Female	Female	Female	Female	Male
No. of fractures	2	1	1	1	1
Cause of fracture	Osteoporosis	Osteoporosis	Osteoporosis	Myeloma	Trauma
Time from fracture to surgery	16-60 days	<15 days	16-60 days	16-60 days	<15 days

**Table 2:** Evaluation of pain with VAS before and after surgery (n=5) Chi-square test p<0.05

Patients	VAS pre-op	VAS after 1 month
Patient 1	08-Oct	03-Oct
Patient 2	09-Oct	02-Oct
Patient 3	10-Oct	03-Oct
Patient 4	10-Oct	02-Oct
Patient 5	10-Oct	02-Oct

No bone cement leakage into the spinal canal, intervertebral disc, or other areas occurred during the procedure. The bone cement volume was 3-4 ml.

## Discussion

The most common surgical techniques for VCF in adults are VP and KP with or without posterior transpedicular stabilization. Historically, the first technique described in the literature was VP by Galibert et al. in the mid-1980s [13], which over time evolved into KP, a safer technique because it allows correction of the fracture and injection of cement at low pressure; the two techniques in principle maintain the same indications. Vesselplasty, which was described later, is a technique that is not very widespread and includes a balloon prosthesis containing cement, making it safer but not very effective in fracture reduction, although each has different advantages and disadvantages.

These techniques aim to strengthen the fractured vertebral body and stabilize the affected level with consequent pain relief. VP achieves a controlled diffusion of cement between the trabeculae of the vertebral body but with a risk of cement leakage to extravertebral compartments (intervertebral disc, soft tissues and paravertebral veins, foramen of conjunction, or even spinal canal) [14-17] and should, therefore, be used in VCFs with an intact posterior wall. Bone cement leakage in the spinal canal can cause a nerve injury with serious consequences. To reduce the risk associated with VP, the KP technique creates a cavity in the vertebral body with an inflatable balloon and then fills the space created with cement, allowing for lower cement injection pressure. This technique, like VP, should be used in vertebral fractures with the posterior wall intact.

Vesselplasty is designed to perform controlled cementation of the vertebral body and minimize cement leakage [18-20] since it contains a prosthesis (balloon or mesh) that contains the cement at the beginning of the injection of cement inside (Vassel-X system), which makes it possible to use it in a vertebra with a compromised or damaged posterior wall. However, it does not reduce the compression fracture, since when the pressure is raised to achieve vertebral remodeling, cement extravasation occurs outside the prosthesis, and the procedure must be stopped due to the danger of cement leakage. The evidence shows that the use of these techniques in vertebral fractures with compromise of the posterior wall of the vertebral body and/or comminuted with more than 50% compromise of their height presents a high risk of cement leakage outside the vertebral body, even invading the spinal canal, which is a contraindication for their use. Xu et al. [21] are one of the few who compared vesselplasty and VP for the treatment of VCF with posterior wall rupture. They found that vesselplasty had a lower bone cement leakage rate, possibly because of the restraint effect of the cement volume.

The technique proposed by the author corresponds to a new variant that combines the two existing techniques, KP plus vesselplasty, to solve this difficulty, thus increasing the surgical indications, especially in elderly patients with comorbidities who often have a high anesthetic risk. In the first stage, a KP is performed to obtain a cavity at the vertebral body level by using a KP balloon. Subsequently, in the second stage, the installation of the Vassel-X system prosthesis is performed with the controlled application of cement. The previously formed cavity allows the application of cement at low pressure, achieving the maximum expansion corresponding to the prosthesis, obtaining a better filling up to the limits of the cavity, and reducing the vertebral fracture with the subsequent cementation safely and effectively (Table 3). Once the initial extravasation is visualized at the limits of the prosthesis under fluoroscopy, the cementation process is stopped to ensure adhesion to the rest of the vertebral body fragments.

**Table 3:** Differences between percutaneous techniques VP: vertebroplasty, KP: kyphoplasty

Technique	Posterior wall	Instrumentation/steps	Risk of leakage
VP	Undamaged (only A1-A2 AO spine fracture)	Cement injection	+++
KP	Undamaged (only A1-A2 AO spine fracture)	1º Balloon insufflation/lifting and removal 2º cement injection	++
Vesselplasty	Damaged (includes A3-A4 AO spine fracture)	1º Mesh insertion, which is inflated through cement injection. The mesh and the cement remain inside the vertebral body	++
Combined technique (KP + vesselplasty)	Damaged (includes A3-A4 AO spine fracture)	1º Balloon insufflation/lifting and removal. 2º Mesh insertion, which is inflated over a previously made cavity, through cement injection. The mesh and the cement remain inside the vertebral body	+

The study has several limitations. The sample size in this study was small, and there is scarce literature comparing the different techniques in patients with vertebral fractures with posterior wall ruptures. However, this variation of the technique showed similar results with respect to postoperative pain, body augmentation, and stability of the vertebra over time, without increasing the cost and risk of local complications, in patients with formal contraindications to traditional cementing techniques. This is made possible by the injection of cement in a prosthesis that contains the cement, preventing its leakage, even though it is a broken vertebra with formal contraindications. Considering the short follow-up time, long-term follow-up is needed.

## Conclusions

Osteoporotic vertebral fracture is a common cause of back pain, dysfunction, and loss of mobility and independence in older adults. These techniques should be used in active fractures, that is, within the first weeks after the injury has occurred, in order to improve pain and stability, as well as to have an effect on preventing kyphosis and thus reducing the associated morbidity and mortality. Percutaneous techniques such as vertebroplasty, kyphoplasty, and vesselplasty have evolved over time. In our center, the combined technique (KP + vesselplasty) allows greater control of cement infusion and thus extends the surgical indication to patients with involvement of the vertebral body and posterior wall (A3-A4, AO spine), which previously presented formal contradictions for traditional techniques. This variation of the percutaneous surgical technique proved to be effective in pain management and the maintenance of normal spine morphology in the series of five cases grouped in our hospital.
